# A Compact Fluorescence System for Tumor Detection: Performance and Integration Potential

**DOI:** 10.3390/bios15020095

**Published:** 2025-02-07

**Authors:** Jean Pierre Ndabakuranye, John Raschke, Preston Avagiannis, Arman Ahnood

**Affiliations:** School of Engineering, RMIT University, Melbourne, VIC 3000, Australia

**Keywords:** biosensor, brain tumor, CMOS color sensors, fluorescence-guided surgery (FGS), miniaturization

## Abstract

Fluorescence-guided surgery (FGS) is an innovative technique for accurately localizing tumors during surgery, particularly valuable in brain tumor detection. FGS uses advanced spectral and imaging tools to provide precise, quantitative fluorescence measurements that enhance surgical accuracy. However, the current challenge with these advanced tools lies in their lack of miniaturization, which limits their practicality in complex surgical environments. In this study, we present a miniaturized fluorescence detection system, developed using state-of-the-art CMOS color sensors, to overcome this challenge and improve brain tumor localization. Our 3.1 × 3 mm multispectral sensor platform measures fluorescence intensity ratios at 635 nm and 514 nm, producing a high-resolution fluorescence distribution map for a 16 mm × 16 mm area. This device shows a high correlation (R2 > 0.98) with standard benchtop spectrometers, confirming its accuracy for real-time, on-chip fluorescence detection. With its compact size, our system has strong potential for integration with existing handheld surgical tools, aiming to improve outcomes in tumor resection and enhance intraoperative tumor visualization.

## 1. Introduction

Brain tumors can be detrimental to patients’ quality of life, with the median survival rate for high-grade tumors ranging from 1 to 3 years [1]. A common form of treatment is craniotomy, which involves surgically removing the tumor. In most cases, a full resection is not feasible due to difficulty distinguishing between healthy and cancerous tissue [2]. Surgeons aim to reduce the chance of cancer recurrence by removing the maximum amount of tumor tissue whilst minimizing the amount of healthy tissue removed [3]. Removing excess healthy brain tissue may result in permanent adverse effects such as motor deficits, dysphasia, increased risk of stroke, and in-hospital mortality [4,5]. This risk can be reduced by utilizing a method to distinguish between healthy and cancerous tissue precisely and accurately. In 1998, Stummer et al. [6] discovered that after exposure to 5-aminolevulinic acid (5-ALA), C6 glioma cells accumulate porphyrin, a naturally occurring body chromophore. Glioma cells metabolize 5-ALA via a disrupted blood–brain barrier and accumulate as protoporphyrin IX (PpIX) [7]. Following the administration of 5-ALA, PpIX fluorophore accumulates at a higher concentration in the tumor tissue compared to the healthy tissue. In surgical settings, this makes it possible to use fluorescence emission to identify the tumor’s high PpIX concentration against the healthy tissue’s background emission, since PpIX fluorophore has a distinct fluorescence signature compared to the tissue autofluorescence [8]. During fluorescence-guided tumor resection surgery, the surgeons use this effect to distinguish between healthy tissue and tumor. Beyond this visual assessment, the quantitative evaluation of the PpIX fluorescence has been shown to allow for more accurate tissue detection. Innately, PpIX glows pink (635 nm peak) under blue light excitation (405 nm) [9], which is the foundation for this application of fluorescence-guided surgery (FGS) for brain tumor removal [10]. The tumor can be detected by monitoring the intensity of the emitted optical signal, with further calculations of this signal indicating the presence of tumor tissue [11].

FGS with 5-ALA-induced PpIX shows great promise and may result in improved tumor resection outcomes but is yet to be fully functional for surgical procedures. An application of this method involves fluorescence imaging microscopes to evaluate the presence and spatial distribution of PpIX during resection surgery [12]. This has been further explored in recent years by developing fluorescence sensing surgical probes to detect the PpIX emission intraoperatively [13]. Although such surgical probes allow for accurate quantitative detection, it is used as a separate surgical instrument, hindering its seamless use during tumor resection since coordinating the probe and tumor resection tool simultaneously can be strenuous for the surgeon, resulting in inaccuracies due to human error. These instruments would be more effective if combined with the resection tool, such as an ultrasonic aspirator, into a single device. Implementing this integrated approach would require miniaturizing the existing fluorescence sensing devices to a millimeter scale. Currently, a large portion of interoperative fluorescence sensing instruments consists of lenses, optical filters, beam splitters and several size-dependent pieces of equipment, as seen in the above cases. This leads to difficulty when attempting miniaturization without negatively impacting the measurement reliability. Alternative sensing devices, such as a spectral sensing chip, may allow for ease of miniaturization without compromising accuracy. The performance of a spectral sensing chip has been previously examined by our team, in which the system achieved a sensitivity of 92.3% and specificity of 98.3% in detecting an ex vivo tumor model at a resolution of 1 × 1 mm^2^ [14].

**Figure 1:**
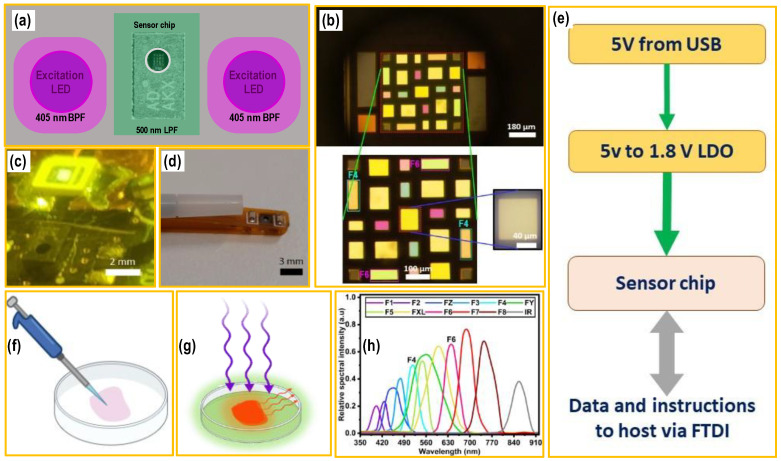
Graphical summary and illustration of the sensing setup: (**a**) Top-view schematics of the sensing system showing the sensor chip, excitation LEDs, and optical filters (bandpass and long pass). (**b**) Photo of the color sensor chip’s CMOS filter mosaics array on top of the silicon photodiode at different magnifications—scale bars: 180, 100, and 40 µm. (**c**) Photo of the fluorescence sensing system with the sensor chip and the LEDs—scale bar: 2 mm. (**d**) Photo of the surgical aspirator’s tip is next to the integrated fluorescence sensing system—scale bar: 3 mm. (**e**) Simplified block diagram of the system. The system is powered by 5 V from a USB through a 1.8 linear dropout voltage regulator. The sensor chip communicates with the host via FTDI. (**f**,**g**) Schematics for the gelatin optical phantom under white light and 395 nm illumination. (**h**) Spectral distribution of the channels available on the sensor chip. Our focus is on F4 and F6.

This work presents a detailed investigation of a miniaturized system for the fluorescence detection of PpIX using an on-chip multispectral sensor, as shown in Figure 1. The device is characterized using a gelatine-based optical phantom model that allows for controllable spectral characteristics. The detection limit of the device is tested by varying optical phantom concentrations of “tumor” regions and comparing it to a conventional benchtop fluorescence detection system. Moreover, the tumor edge detection accuracy is reported and compared to the high-accuracy benchtop spectral system.

## 2. Materials and Methods

### 2.1. Measurement System Characterization

#### 2.1.1. Optical Setup

Optical sources (LEDs) and color filters are vital to the measurement and setup—the simplified system diagram is shown in Figure 1e. Consistent with the literature, a 395 nm LED (MPN: 153283407A212, Würth Elektronik GmbH & Co. KG, Niedernhall, Germany) was used as an excitation source. However, violet LEDs present a spectral shoulder beyond 500 nm, which would be detrimental to our fluorescence measurements if not accounted for. The AF intensity of the brain is very dim, making it susceptible to interference with the excitation LED spectral shoulder. To counter this, a 405 nm bandpass filter (BPF) (PN: 15–117, Edmund Optics, Singapore) was used on top of the excitation source to cut off over one order of magnitude of the spectral shoulder. Furthermore, light emitted from the sample must be separated from the excitation source. Thus, a 425 nm long pass filter (LPF) (PN: 84–736, Edmund Optics) was used with the detector to remove the 385–415 nm spectral component. The spectral distribution and response of the excitation nm LED, shoulder, and optical filters are shown in Figure S1.

A benchtop system illustrated in Figure S2a was built to assess the feasibility of fluorescence measurement on optical brain phantoms and used to evaluate the multispectral chip. The setup consisted of the chip at its center and a spectrometer as a validation tool. It also incorporates two flanking excitation LEDs covered by bandpass optical filters. The LED-chip sensing system was configured to reduce optical shunting, optimizing the collection of the fluorescence signal, and was kept intact using a retort stand. A movable XY-stage was used to hold the gelatine model and was clamped to the poles screwed onto the optical breadboard. This was carried out to mitigate errors due to vibration-induced irregularities, motion artifacts and geometrical variabilities. Note that a nonconductive material was used to avoid potential shorting.

#### 2.1.2. Electronic Hardware and Software

***Full range spectral measurement—USB2000+ spectrometer:*** The spectral fluorescence measurements were carried out using a spectrometer (USB2000, Ocean Insight, Inc., Orlando, FL, USA), and a fiber optic cable (R400-7VIS-NIR, Ocean Insight, Inc.). OceanView 2.0 software (Ocean Insight, Inc.) was used to capture the spectral data from the spectrometer, which has the additional features of setting spectral smoothing parameters (average of 50 scans) and integration time (10 ms). Since the spectral measurement with the spectrometer provides a high degree of accuracy, it was used to validate the on-chip measurements.

***Multi-spectral sensing chip:*** The multispectral chip was evaluated using a system with a simplified block diagram in Figure S2b and was hosted via an I^2^C-FTDI interface by an FT232H I2C to USB converter. The board was evaluated using EvalSW_ALS v1 (ams OSRAM AG, Premstaetten, Austria) application to provide control of the sensor registers. Unlike most other systems where the sensor’s GPIOs are indirectly accessed by hosting it using a microcontroller unit, an FT232H chip is used as an I2C to USB converter. Coupled with an API, the EvalSW allows for computer-controlled direct access to the sensor’s GPIOs. The sensor is programmed to relay the raw data to the computer via a USB and export it in an Excel file for later analysis. Data are collected as ADC counts following the conversion of photocurrents by a 16-bit resolution ADC.

The hardware was further improved by integrating optics to generate an endoscopic window made of diamond. The sensor is intended to be used for proximity measurements. As such, the risk of brain tissue adhesion onto the sensor must be minimized. A diamond window was previously characterized and found to have lower adhesion than a glass slide [14]. The diamond surface performed better than a glass coverslip, and the latter was attributed to its hydrophobic surface.

### 2.2. Brain and Brain Tumor Optical Phantoms

We prepared a brain optical phantom using off-the-shelf ingredients, including bovine gelatine powder and fluorescent dye-based inks. Our recipe mimics the brain tissue’s fluorescence properties without alluding to absorption, scattering, and mechanical properties.

The phantoms were prepared by sprinkling 2 g of food-grade gelatine powder (McKenzie’s Food, Melbourne, VIC, Australia) to 10 mL of room temperature water in a quartz vial, then gently mixed to avoid introducing air bubbles. Water was poured into a beaker and heated to approximately 60 °C using a hotplate. The quartz vial was then sealed and placed into the warm water bath in the beaker to completely melt the gelatine in the quartz vial until a homogenous mixture was achieved. At this stage, the gelatine mixture was still highly concentrated, and subsequent dilution was carried out by gently adding the concentrated gelatine mixture into warm water in a Schott bottle and continuously stirring to achieve a 4 g/dL gel-like solution. Gelatine alone does not have detectable AF properties in the spectrum of interest (500–700 nm) under 405 nm excitation [15]. To provide fluorescence, two samples were created by first adding 15 mL of the stock gelatine solution into two separate vials. In the first vial, 5 μL of green ink (Anko, Melbourne, VIC, Australia) was added to mimic the healthy brain tissue autofluorescence. In the second vial, 5 μL of pink ink (Anko, Australia) was added to the vial to imitate the fluorescence of PpIX.

The “tumor”-in-“brain” optical phantom model was created by filling the green-inked gelatine solution (healthy brain phantom) into a chambered coverslip (µ-slide well, Cat.No:82106, Ibidi, Gräfelfing, Germany) and laying ~4 µL of the pink-inked gelatine solution (PpIX phantom) on top. This allowed diffusion-driven mixing between the PpIX and brain phantom to create a tumor–brain fluorescence signature. The mixing by diffusion was stopped by placing the µ-slide well into a refrigerator at the temperature of 5 degrees Celsius to solidify the samples—Figure S3a.

### 2.3. Measurement Procedure

In all cases, the two inks were mixed to produce a maximum ratio value of 5 at the center of the tumor phantom [12] using Equation (1), which is an extension of Equation (S1) (SI) with zero I_background_ [16]. This is commonly known as “ratiometric approach” and is utilized in several other areas of analytical biochemistry to optically detect analytes [17]. The numeric values of ratios obtained using the color sensor chip will differ from those obtained using the spectrometer. This is primarily since the color filters on the chip have full width at half maximum (FWHM) values of 42 nm (F4) and 50 nm (F6)—see Figure S1. Given that the spectrometer offers a resolution of about 0.5 nm, this means that the sensor measures light intensity over a broader range of wavelengths. Although this difference affects the calculated ratio, and integrating intensity across the entire spectral range would be ideal Equation (S2), the clear separation in spectral properties allows for reliable differentiation between autofluorescence and PpIX, rendering the high spectral resolution of the spectrometer unnecessary in this context.

(1) $$Ratio=\frac{I_{635\;nm}}{I_{514\;nm}}$$

The tumor and tumor margins were detected using the spectrometer and the sensor chip, as described in Figure S2. The multispectral chip and the tumor model were separated by ~4 mm, with a typical optical power of 100 μW. The separation was chosen to deliver the optimal spectral fluorescence intensity from the brain phantom. For accurate positioning, the sample was mounted on a movable XY stage.

Measurements on the multispectral chip were taken in emissive mode, and the experiments were performed in a darkened room where the only light source was from the excitation source to prevent optical interference. In the case of spectrometer measurements, the optical fiber was mounted perpendicularly to the bench to capture light from the sample in specular mode (non-diffuse) at 90 degrees. This allowed optimal intensity reading and minimized any refraction or reflection by angled light. This is required as the fiber’s numerical aperture is ~10 degrees.

## 3. Results and Discussions

### 3.1. Optical Phantom Characteristics

**Figure 2:**
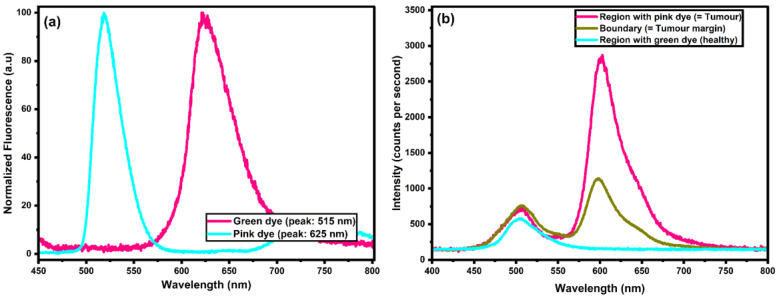
(**a**) Normalized spectral distribution of fluorescence for ink as surrogates of normal tissue (cyan) and tumor (pink). (**b**) Spectral distribution of the optical brain-tumor phantom at different spots, including tumor region, tumor margin, and healthy tissue.

The optical phantom produced fluorescence in the peaks of interest (515 nm and 625 m), as seen in Figure 2a. The chosen inks have autofluorescence (AF) peaks at 515 and 625 nm to mimic the brain’s AF and PpIX fluorescence, respectively. Although these peaks slightly deviate from the brain AF and PpIX emissions (510 nm and 635 nm), this difference is inconsequential, as the sensor cannot resolve the small variations in the spectrum. As shown in Figure 2b, the ink concentrations were successfully adjusted to provide the fluorescence profiles that mimic those of the tumor tissue, as reported in high-grade tumor (HGT) patients by, with a maximum ratio of 4.8 Richter, Haj-Hosseini [12]. This is confirmed by the 1:4 ratio obtained from the AF peak (I_AF_) to the PpIX peak (I_PpIX_) ratio in both Figure S4 and Figure 2b.

It should be noted that there are differences between the phantom and tissue fluorescence profiles outside the major peaks. This is due to other contributing factors to the brain tumor profile, which have not been accounted for here. These include other chemical compounds in the brain, such as Lipofuscin and NADH [18], blood leakage from the blood–brain barrier [19], the photodegradation of PpIX and AF, and brain mechanical properties. Though these properties were not considered, the fluorescence for the desired peaks and correlated channels was assessed, and no photo decay was observed throughout the experiment.

### 3.2. Fluorescence Detection

**Figure 3:**
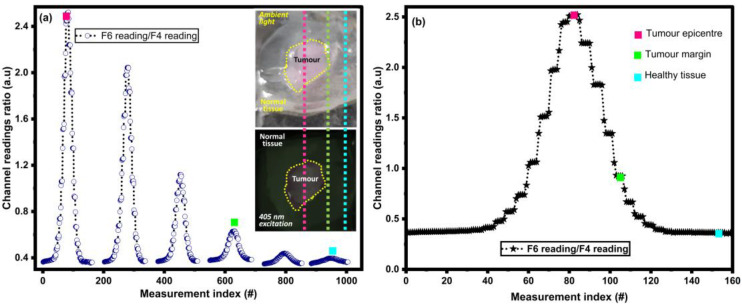
Spatial distribution sensor chip data (**a**) at intermittent lateral spots (yellow dotted vertical lines) and (**b**) through the tumor epicenter.

The XY spatial scanning procedure was performed to detect the varying concentrations across the sample and confirm the edge detection. Both were successfully detected with the spectrometer and the multispectral chip, with the results from the multispectral chip depicted in Figure 3. The reduction in fluorescence intensity, correlated with a lower concentration, in the outer sections of the tumoral region is shown in Figure 3a. We used this spatial variation in the concentration to benchmark the multispectral chip against a spectrometer over a range of concentration values.

A ratio of the F6 channel to the F4 channel was used for fluorescence detection, a variation in the ratio commonly used in this practice. This ratio is usually taken as the fluorescence intensity at 635 nm (I_PpIX_) minus the expected intensity of healthy brain tissue at 635 nm (I_AF@635nm_), divided by the fluorescence intensity at 514 nm (I_AF_) [20] (as the fluorescence at 510 nm should be consistent across the brain tissue)—see Equation (S1). This ratio allows for a relative measurement across different brain sections and accounts for minor variations in lighting intensity. As there is no shoulder from the green dye at 635 nm, as shown in Figure 2, the value for I_AF@635nm_ is 0. Hence, the ratio of I_PpIX_ divided by I_AF_ was used for our analysis and correlated to F6/F4.

Using this ratio, the lower limit to reliably confirm that the detection of brain tumor tissue within brain samples was reported as ≥0.6 [12]. The lowest ratio detected from our sample was approximately 0.4, as shown in Figure 3, implying that the multispectral chip is sensitive enough to reliably detect the lower limit for tumors. With this high level of sensitivity, the lower limit for the ratio may be further reduced, improving the amount of tumor tissue detected.

### 3.3. Limit of Detection

**Figure 4:**
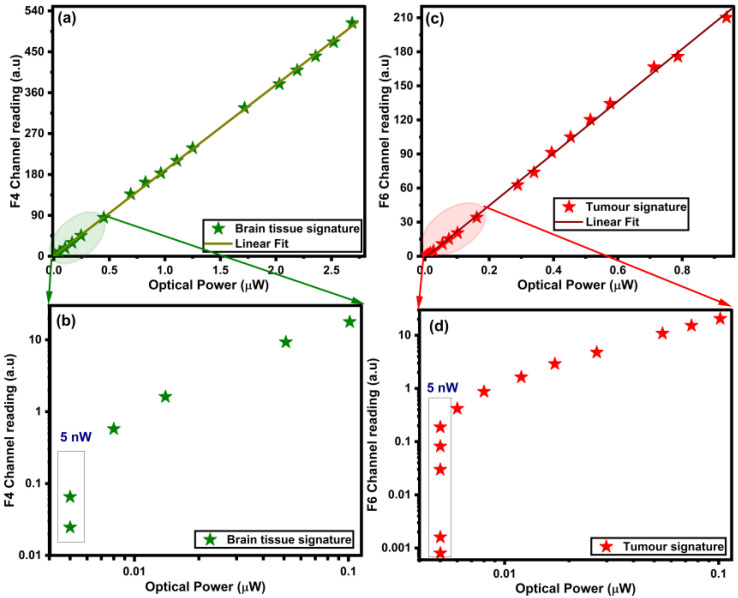
Comparison of the optical power measurements and the ADC counts using (**a**,**b**) green and (**c**,**d**) red optical sources shown in linear (**a**,**c**) and logarithmic (**b**,**d**) scales.

The spectral calibration of the multispectral chip was achieved by comparing the optical power measured by the FieldMax II Laser Power Meter (Coherent Inc., Santa Clara, CA, USA) and the collected ADC counts, as shown in Figure 4. As highlighted in Figure 4a,b, there is a linear relation between the light intensity as measured using the power meter and the sensor chip’s output. Moreover, the chip measurement showed an even higher sensitivity than the power meter at low light intensities. As shown in Figure 4c,d, the sensor chip was capable of detecting three orders of magnitude variations in the light intensity at optical powers of below 5 nW on the red channel. Similarly, the chip detected over one order of magnitude variations in the light intensity in the green channel. At intensities of less than 5 nW, the optical power meter could not detect the changes in the light intensity. This high sensitivity implies that the sensor can provide a high level of accuracy when determining the presence of small PpIX quantities. Furthermore, it may allow low excitation intensities, which mitigates the photobleaching challenge [21].

### 3.4. Spectral Selectivity

The selectivity study was carried out to investigate how discriminative the sensor is. Here, the idea is if one shines light with the brain’s AF signature (i.e., a green light) on the chip, how much could be detected on the F4 channel (515 nm) compared with the F6 channel (635 nm), and if we shine the light with the tumor’s fluorescence signature (i.e., a red light), how much could be detected on the F6 channel (635 nm) compared to the F4 channel (515 nm). This study was performed using both the spectrometer and the multispectral chip. The selective ability of the sensor and the spectrometer was obtained using a measure of percentage contrast error—Equation (2).

(2) $$Contrast\;error=\left\{\begin{array}{c} \;\;\;\;\;\;\frac{100\times Intensity\left(514\;or\;635\;nm\right)}{Intensity\left(514\;nm\right)+Intensity\left(635\;nm\right)}\\ \frac{100\times Channel\;reading\;(F4\;or\;F6)}{F4\;channel\;reading\;+\;F6\;channel\;reading} \end{array}\right.$$

**Figure 5:**
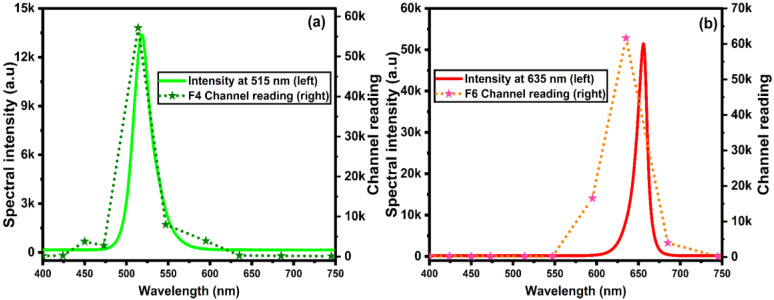
Illustration of the sensor’s ability to distinguish between healthy tissue and tumor by measuring the (**a**) error induced on the F6 channel when exposed to the “healthy tissue fluorescence signature” and (**b**) the error induced on F4 channel when exposed to the “tumor fluorescence signature”.

This analysis aims to evaluate the performance of the chip system in distinguishing between healthy and tumor tissues. To assess this, we measured the error on the “tumor channel” to a healthy cell’s fluorescence signature (green LED) and the error on the “healthy channel” when exposed to the tumor tissue’s fluorescence signature. As shown in Figure 5a,b, there is a clear separation between the two, with a calculated contrast of over 99% for both tumor and brain signatures. This separation ranges over two orders of magnitude, suggesting that the chip has high selectivity, and it could be used to measure the ratio given in equation (2). As shown in Table 1, the chip system also shows a low error rate of less than 0.5%. Moreover, this works even at low fluorescence intensities, where human perception typically performs poorly in distinguishing between colors [22].

### 3.5. Performance Evaluation and Study Limitations

**Figure 6:**
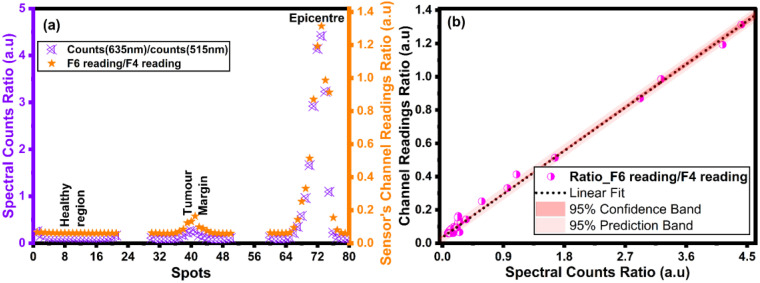
(**a**) The sensor chip and the spectrometer data measured on the optical tissue phantom at three distinct spots. The spectrometer output (counts per second) at 635 nm and 515 nm are used to calculate the spectral counts ratio. (**b**) Correlation analysis of the sensor chip and the spectrometer.

The validation studies were performed to compare the full-range spectral measurements and on-chip measurements. The readings obtained from both measurement procedures were aligned with the same section of the sample. These were aligned at the regions of healthy tissue, tumor margin, and tumor epicenter—Figure 6a. Correlation and regression analyses were used by comparing the ratios of the same sections, which resulted in an R-squared of approximately 0.98—Figure 6b. This shows that the sensor chip could perform at a similar sensitivity as the spectrometer. This high level of sensitivity, combined with the small dimensions of the chip, makes it ideal for designing a combined resection and detection tool to be used in FGS with 5-ALA.

On the other hand, several studies based on fluorescence-guided surgery (FGS) have been reported. Table 2 provides a summary and comparison of recent studies. While these studies highlight benefits such as high accuracy, sensitivity, and specificity, the miniaturization required for potential integration into tumor resection procedures remains a challenge. Our study outperforms these in this regard.

The measurements in this study were taken with the optical phantom, which, from Figure 2a, shows more distinct peaks than brain tissue with a tumor, as seen in Figure S4. This gives the advantage of testing the chip’s ability to detect specific peaks but does not consider the chip’s ability to perform this detection with additional interferences. To ensure the effective use of such devices, further work will need to be performed to ensure they can reliably detect these fluorescence quantities with a sample containing brain tissue and brain tumor tissue with increased PpIX concentration.

## 4. Conclusions

This study investigated the feasibility of the in situ detection of tumor tissue with a miniaturized sensor during resection. This was achieved by developing and validating an effective optical brain phantom from gelatine and ink. An on-chip multispectral sensor in a 3.1 × 2 × 1 mm package provided quantitative detection of PpIX emission relative to background AF at varying concentrations. This was achieved with a sensitivity of about 98% compared to a highly accurate spectrometer measurement. This suggests that the tumor margin can be detected, allowing for the distinction between the regions of the brain with and without tumor tissue. The hardware was improved by incorporating an endoscopic window made of diamond, improving the performance due to its hydrophobic surface. This shows promising results for integrating the hardware into a handheld resection tool such as an ultrasonic aspirator. The miniature sensing platform reported in this work readily meets the denominational constraints of ultrasonic aspirators, such as the SONOPET system, with an inner tip diameter of 1.5 mm [21]. Such a smart surgical resection tool may allow the surgeon to distinguish between healthy and tumor tissue more reliably during the surgery itself. This could improve the success of craniotomy surgeries and reduce the risk of tumor recurrence, increasing the survival rate of brain tumor surgeries. In this work, we have only focused on PpIX, but indocyanine green (ICG) is also an important FDA-approved marker with emission in the 800~850 nm range, which makes it possible to detect using our proposed miniaturized solution. Further testing on tumor samples induced with 5ALA will need to be performed to confirm these results.

## Figures and Tables

**Table 1:** Detection selectivity of the chip system for distinguishing between healthy and tumor fluorescence in an optical tumor phantom.

	Healthy Sensor Channel (515 nm)	Tumor Sensor Channel (635 nm)
Green LED (healthy tissue’s fluorescence signature)	57,169 (99.6%)	212 (0.4%)
Red LED (tumor tissue’s fluorescence signature)	279 (0.5%)	61,653 (99.5%)

**Table 2:** Comparison table of competing fluorescence guided surgery-based techniques for glioma detection.

Study	Dye Detected	Excitation Mode	Method of Detection	Benefit	Reported Values	Limitation
This study	Ink dyes in optical phantoms	Singe mode FGS (405 nm)	Multispectral sensing using CMOS 514 nm and 635 nm color filters.	Highly miniaturized.	R^2^ > 0.98 compared with benchtop spectrometers.	Experiments performed with inks to imitate fluorescence profiles
Ndabakuranye, Belcourt, 2024 [14]	PpIX in ex vivo rat brain	Single mode FGS (405 nm)		Highly miniaturized and incorporates an endoscopic window.	Sensitivity of 92.3% and a specificity of 98.3%.	Device cannot be tuned to detect other wavelengths
Black, Kaneko, 2021 [18], Black, Byrne, 2024 [23]	PpIX	Hyperspectral FGS	Hyperspectral measurement using 620 nm and 634 nm peaks.	Improved sensitivity and precision of PpIX quantification.	85.7% accuracy for glioma margin classification.	Long recording and data processing time
Kulkarni, Reed, 2024 [24]	ICG and PpIX	Dual mode FGS	Based on fiber optic measurement.	Improved accuracy due to multivariate analysis.	In-depth detection of 20 mm.	Used on pancreatic adenocarcinoma tumors, not gliomas
Dolganova, Varvina, 2022 [25]	PpIX in ex vivo rat brain	Single mode FGS	Based on sapphire scalpel.	Hard and strong tip. Can be used with other fluorophores (rhodamine 6 G).	2 mm resolution.	Feasibility test only, further engineering work needed to meet requirements for surgical oncology
Bravo, Olson, 2017 [26]	PpIX in vivo	Hyperspectral FGS	Spectral fitting, filtering, and optical correction.	Larger region can be analyzed at a high accuracy.	Limit of detection of 0.014–0.041 µg/ml.	Requires data processing after imaging
Richter, Haj-Hosseini, 2017 [12]	PpIX in vivo	Singel mode FGS	Fluorescence spectroscopy-based handheld probe.	Region specific analysis, accounting for background signal (AF).	For fluorescence ratios >0.6, specificity is 0.66.	Requires separate probe for operation
Lovato, Araujo, 2017 [27]	Fluorescein	Single mode FGS	Attachment made for “any microscope”.	Cost effective “total cost to build our device is €340”.	Reported sensitivity of 79–97% and specificity of 81–100% for fluorescein. Not device specific.	Performance of device not discussed

## Data Availability

Data is contained within the article or Supplementary Materials.

## Supplementary Materials

The following supporting information can be downloaded at: www.mdpi.com/article/10.3390/bios15020095/s1, Figure S1: (a) Spectral distribution of the Bandpass and long pass optical filter responses. (b) Excitation source (both with and without the shoulder removal filter). The Light Emitting Diode (LED) has a spectral peak at 395 nm; however, due to the spectral response of the pass-band optical filter at 405 nm, the peak is shifted accordingly. (c) Spectral responsivities for F1, F4 and F6 channels; Figure S2: (a) Schematic illustration of the sensing setup. (b) Simplified block diagram of the system. (c) Photo of the colour filters on top of the silicon detector arrays; Figure S3: (a) Photo of the optical phantom placed in an ibidi-chambered coverslip. The inset photo major spatial scanning lines during the sensor evaluation. (b,c) Photo of the brain-tumour optical phantom with tumour margins and regions taken under (a) ambient illumination and (b) Excitation light. Note that (c) is captured using an optical filter. The red dotted line represents the edge (margin) of the tumour region; Figure S4: A typical spectral fluorescence intensity distribution of PpIX, both as a (a) standalone chromophore and (b) in-tissue chromophore. The green dotted line represents the emission distribution of healthy brain tissue; Table S1: Electrical and optical characteristics of the multispectral chip.
