# Supplementary material for: A Compact Fluorescence System for Tumor Detection: Performance and Integration Potential
Source: Biosensors (Basel). 2025 Feb 7;15(2):95. doi: 10.3390/bios15020095 (PMC11853339; doi:10.3390/bios15020095)
Supplement: Supplementary file 1 [file biosensors-15-00095-s001.zip › biosensors-3380059-supplementary.pdf]

SUPPLEMENTARY INFORMATION

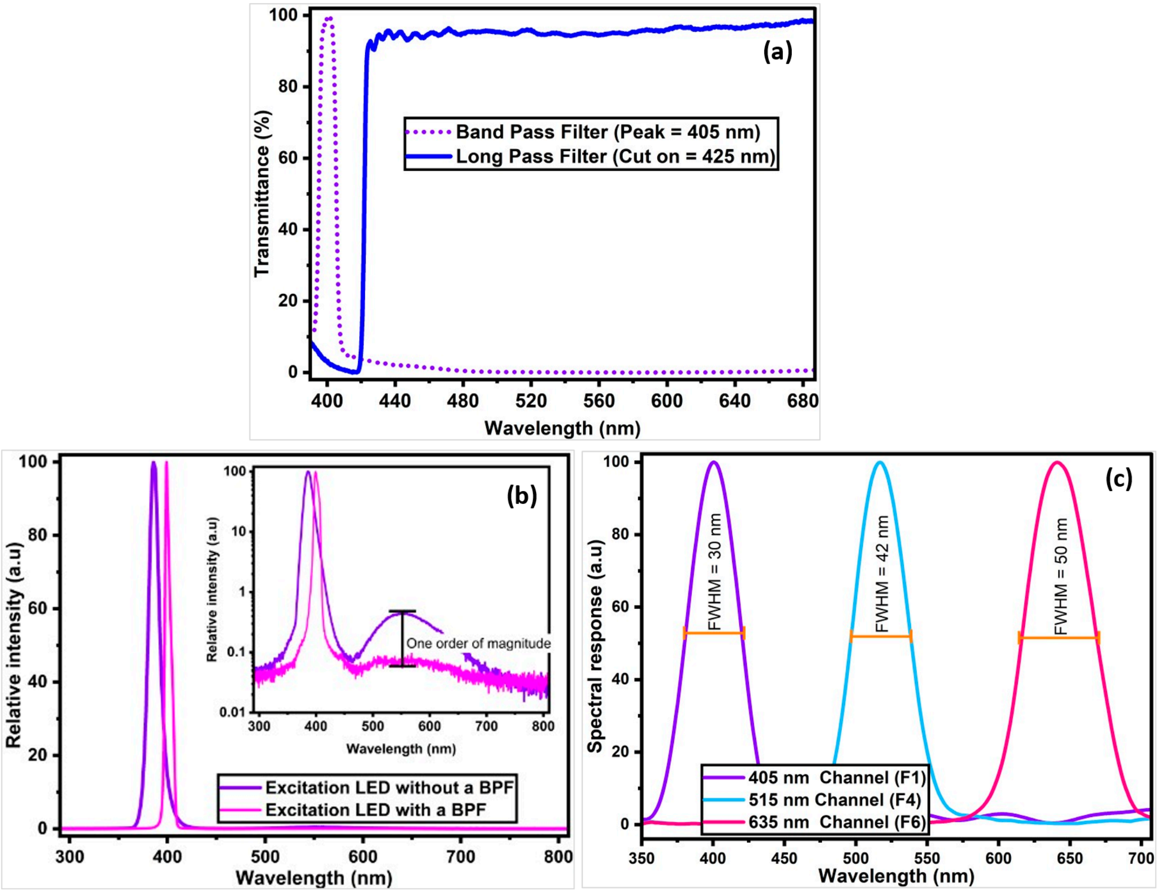

Figure S1. (a) Spectral distribution of the Bandpass and long pass optical filter responses. (b) Excitation source (both with and without the shoulder removal filter). The Light Emitting Diode (LED) has a spectral peak at 395 nm; however, due to the spectral response of the pass-band optical filter at 405 nm, the peak is shifted accordingly. (c) Spectral responsivities for F1, F4 and F6 channels.

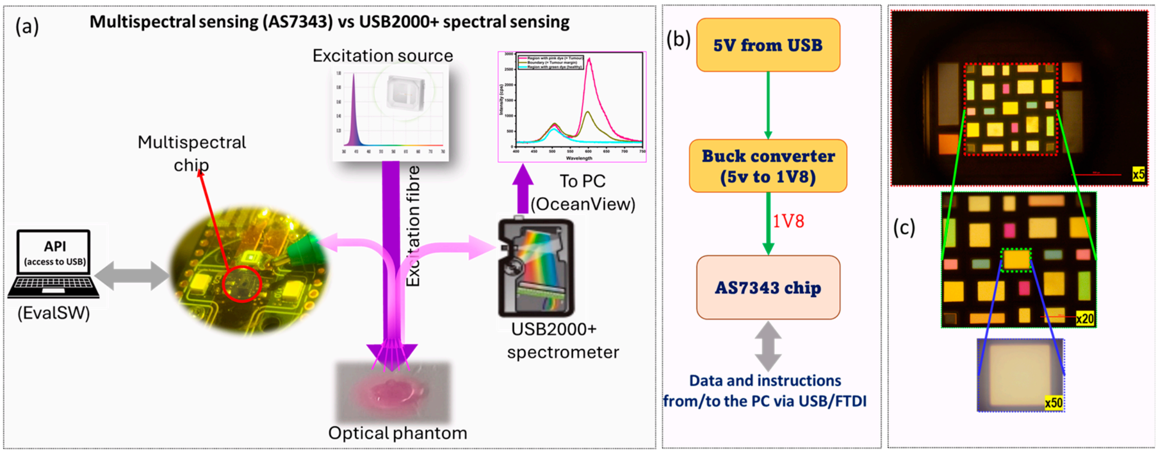

Figure S2. (a) Schematic illustration of the sensing setup. (b) Simplified block diagram of the system. (c) Photo of the colour filters on top of the silicon detector arrays.

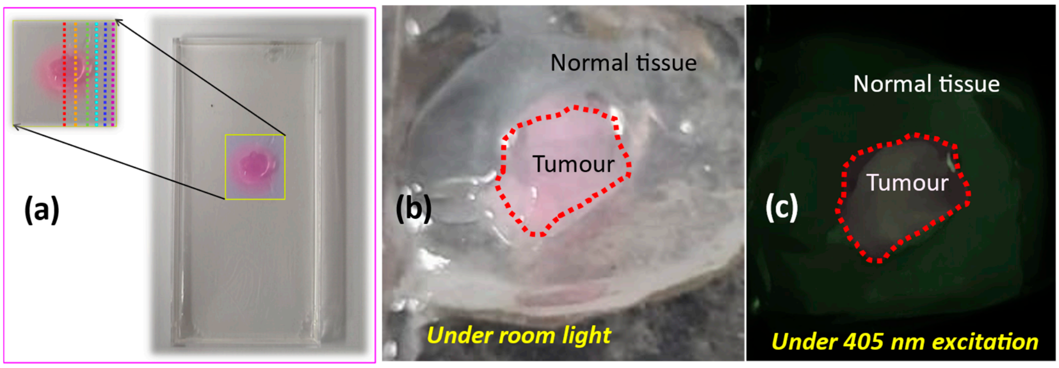

Figure S3. (a) Photo of the optical phantom placed in an ibidi-chambered coverslip. The inset photo major spatial scanning lines during the sensor evaluation. (b,c) Photo of the brain-tumour optical phantom with tumour margins and regions taken under (a) ambient illumination and (b) Excitation light. Note that (c) is captured using an optical filter. The red dotted line represents the edge (margin) of the tumour region.

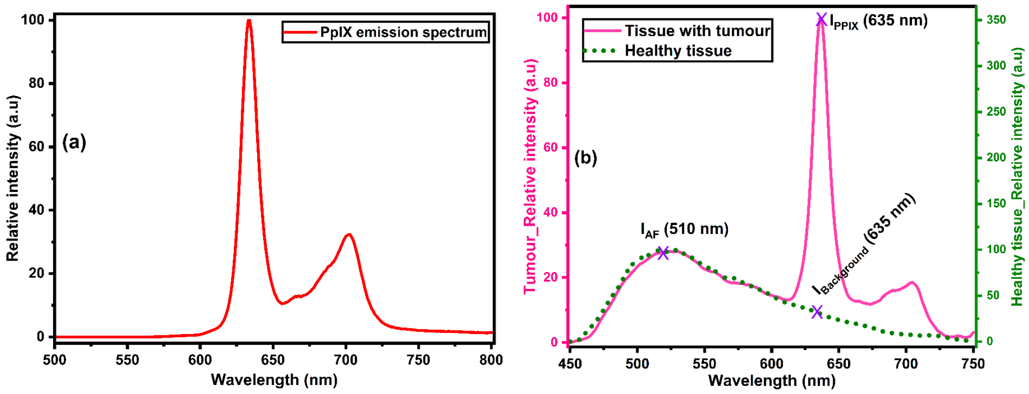

Figure S4. A typical spectral fluorescence intensity distribution of PpIX, both as a (a) standalone chromophore and (b) in-tissue chromophore. The green dotted line represents the emission distribution of healthy brain tissue.

Details About the Multispectral Sensor (AS7343)

AS7343 (ams OSRAM., Munich, Germany) is a miniature multispectral sensor for spectral and colour measurement over fourteen optical channels. Of the fourteen, eleven channels are distributed over the visible range, one near-infrared, and one clear and flicker detection channel. The visible range channels included those with a peak wavelength of 405 nm (F1), 515 nm (F4), and 635 nm (F6), whose spectral responsivities are depicted in Fig. S1 (c). These aligned well with the spectrums of interest for the experiment. The chip is housed in a low-profile OLGA-8 package (3.1 x 2 x 1 mm) and is designed to operate in transmissive, reflective, and emissive modes. Table S1 summarises several optoelectronic characteristics.

Table S1. Electrical and optical characteristics of the multispectral chip.

Parameter	Conditions	Value (Range)
Supply voltage (V_{DD})	-	1.8 V (1.7 - 1.98 V)
Supply current (I_{DD})	Active mode (V_{DD} = 1.8 V)	210 µA (max = 280 µA)
	Idle mode (V_{DD} = 1.8 V)	40 µA (max = 60 µA)
	Sleep mode (V_{DD} = 1.8 V)	0.7 µA (max = 5 µA)
Spectral channels	F1 (λ_{max} = 405 nm, $\Delta\lambda_{½}$= 30 nm), F4 (λ_{max} = 515 nm, $\Delta\lambda_{½}$= 42 nm), F6 (λ_{max} = 635 nm, $\Delta\lambda_{½}$= 50 nm)	

Optical Properties of Brain Tumours

Under blue light excitation (395 nm), the spectral characteristics of healthy brain tissue and common PpIX-containing brain tumours have spectral peaks at 510 and 635 nm, respectively – Fig. S4. Based on these spectral signatures, a quantitative approach has been used value correlating to tumour presence can be obtained from Eq. S1 [1]. This ratio provides a relative comparison between the spectral fluorescence intensity of PpIX (635 nm peak) and the brain's AF intensity (510 nm peak). This ratio provides a direct correlation for tumour presence, with a higher ratio indicating a higher PpIX concentration; thus, the tumour malignancy [2]. Using a ratio allows for the detection to be performed under slightly different lighting environments.

$$Ratio = \frac{I_{635\,nm} - I_{background}}{I_{510\,nm}} \qquad \text{(S1)}$$

As shown in SI Fig.1, the ratio in equation (1) is expected to vary due to differences in spectral distribution, with F4 and F6 having FWHM values of 42 nm and 50 nm, respectively—about two orders of magnitude larger than the 0.5 nm resolution for USB2000 spectrometer. Additionally, figure S4 shows the autofluorescence peak at 510 nm (FWHM: 118 nm) and PpIX fluorescence at 635 nm (FWHM: 14 nm). Figure 1(h) further indicates that F4 has 5.8% lower spectral responsivity than F6, suggesting that the sensor will respond less to autofluorescence. While integrating intensity over the entire spectral range (Eq. S2) is ideal, the clear spectral separation between autofluorescence and PpIX makes the spectrometer's high resolution redundant for our application.

$$\frac{\sum_{\lambda=400\,\mathrm{nm}}^{750\,\mathrm{nm}} \mathrm{Fluorescence}(\lambda)_{\mathrm{tumour}} * \mathrm{Responsivity\ (F6)}}{\sum_{\lambda=400\,\mathrm{nm}}^{750\,\mathrm{nm}} \mathrm{Fluorescence}(\lambda)_{\mathrm{tissue}} * \mathrm{Responsivity\ (F4)}} \qquad \text{(S2)}$$

Bibliography

[1] N. Bendsoe *et al.*, "Fluorescence monitoring of a topically applied liposomal Temoporfin formulation and photodynamic therapy of nonpigmented skin malignancies," *Journal of*

Environmental Pathology, Toxicology and Oncology, vol. 26, no. 2, 2007, doi: https://doi.org/10.1615/jenvironpatholtoxicoloncol.v26.i2.60.
[2] J. C. Richter, N. Haj-Hosseini, M. Hallbeck, and K. Wårdell, "Combination of hand-held probe and microscopy for fluorescence guided surgery in the brain tumor marginal zone," *Photodiagnosis and photodynamic therapy,* vol. 18, pp. 185-192, 2017, doi: https://doi.org/10.1016/j.pdpdt.2017.01.188.
